# Improved Itaconate Production with *Ustilago cynodontis* via Co-Metabolism of CO_2_-Derived Formate

**DOI:** 10.3390/jof8121277

**Published:** 2022-12-05

**Authors:** Lena Ullmann, Nils Guntermann, Philipp Kohl, Gereon Schröders, Andreas Müsgens, Giancarlo Franciò, Walter Leitner, Lars M. Blank

**Affiliations:** 1 iAMB—Institute of Applied Microbiology, ABBt—Aachen Biology and Biotechnology, RWTH Aachen University, Worringerweg 1, 52074 Aachen, Germany; 2 ITMC—Institute for Technical and Macromolecular Chemistry, RWTH Aachen University, Worringerweg 2, 52074 Aachen, Germany; 3 Max Planck Institute for Chemical Energy Conversion, Stiftstraße 34-36, 45470 Mülheim an der Ruhr, Germany

**Keywords:** itaconate, itaconic acid, CO_2_ hydrogenation, secondary metabolites, Ustilaginaceae, *Ustilago cynodontis*, DoE, inverted biphasic catalysis, formate, *Ustilago*

## Abstract

In recent years, it was shown that itaconic acid can be produced from glucose with *Ustilago* strains at up to maximum theoretical yield. The use of acetate and formate as co-feedstocks can boost the efficiency of itaconate production with Ustilaginaceae wild-type strains by reducing the glucose amount and thus the agricultural land required for the biotechnological production of this chemical. Metabolically engineered strains (*U. cynodontis* Δ*fuz7* Δ*cyp3* ↑P_ria1_ and *U. cynodontis* Δ*fuz7* Δ*cyp3* P_etef_*mttA* ↑P_ria1_) were applied in itaconate production, obtaining a titer of 56.1 g L^−1^ and a yield of 0.55 g_itaconate_ per g_substrate_. Both improved titer and yield (increase of 5.2 g L^−1^ and 0.04 g_itaconate_ per g_substrate_, respectively) were achieved when using sodium formate as an auxiliary substrate. By applying the design-of-experiments (DoE) methodology, cultivation parameters (glucose, sodium formate and ammonium chloride concentrations) were optimized, resulting in two empirical models predicting itaconate titer and yield for *U. cynodontis* Δ*fuz7* Δ*cyp3* P_etef_*mttA* ↑P_ria1_. Thereby, an almost doubled itaconate titer of 138 g L^−1^ was obtained and a yield of 0.62 g_itaconate_ per g_substrate_ was reached during confirmation experiments corresponding to 86% of the theoretical maximum. In order to close the carbon cycle by production of the co-feed via a “power-to-X” route, the biphasic Ru-catalysed hydrogenation of CO_2_ to formate could be integrated into the bioprocess directly using the obtained aqueous solution of formates as co-feedstock without any purification steps, demonstrating the (bio)compatibility of the two processes.

## 1. Introduction

Itaconic acid is an attractive bio-based building-block that has the potential to act as a green substitute for many petroleum-based chemicals in the polymer industry [1]. It is an unsaturated dicarboxylic acid with two carboxy and one methylene group. The presence of these functionalities, along with a conjugated double bond, makes itaconic acid a versatile platform chemical for multiple applications, such as styrene-butadiene rubbers, synthetic latexes, super absorbents, unsaturated polyester resins, plastics, coatings, chemical fibers, biofuels and detergents [1].

For the last 70 years, itaconic acid has been produced using *Aspergillus terreus*, reaching titers of 130 g L^−1^ [2,3]. However, various drawbacks are associated with the use of this native producer. For instance, it shows filamentous growth and its itaconate production is morphology-dependent. Further, it leads to comparably low yields (<0.50 g_itaconate_ g_glucose_^−1^, i.e., 69% of the maximum theoretical yield) in combination with long fermentation times (>150 h), it is sensitive to shear forces and interruption of oxygen supply [2]. All these factors limit the process efficiency and increase production costs. Indeed, the comparably high price, close to 2 USD/kg, holds back the broader commercial application of itaconic acid [4]. To circumvent these limitations, Ustilaginaceae are sought as alternative itaconic acid production hosts, with a special focus on *Ustilago maydis.* This non-filamentous native producer could be suitable for industrial production as it shows the highest reported titer at laboratory scale so far (220 g L^−1^). The increased titer was obtained through integrated metabolic and morphological engineering, accompanied by process optimization and in-situ crystallization of itaconic acid as calcium itaconate [5]. Metabolic engineering focused on targets such as the overexpression of the mitochondrial transporter Mtt1, the overexpression of the cluster-associated regulator Ria1, disrupting the itaconate oxidase encoding gene *cyp3*, as well as heterologous expression of the mitochondrial transporter MttA from *A. terreus* [6,7]. Furthermore, deletion of *fuz7* enables a stable yeast-like growth [8]. Moreover, a metabolomics method focusing on the central carbon metabolism has recently been developed for *U. maydis*, which can be applied to investigate the cellular metabolic network and support metabolic engineering strategy [9].

Furthermore, the morphology of *Ustilago* can be controlled by deletion of the *fuz7* gene, abolishing filamentous growth and stabilizing the yeast-like morphology [8].

During previous studies, significant improvements by strain- and process engineering were achieved, and *Ustilago* strains are now available producing itaconic acid at almost maximum theoretical yield [10]. With the awareness that glucose and other carbohydrates compete for valuable resources of agricultural land, much effort has been dedicated to the search of co-feedstocks able to improve the overall process efficiency. Recently, C1 compounds have received attention as microbial co-feedstocks as they can be readily produced out of widely available resources such as carbon dioxide utilizing renewable energy (“power-to-X” technologies). Formate, in particular, can be generated effectively from CO_2_ via photochemical or electrochemical reduction [11,12,13,14,15] or by catalytic hydrogenation [16,17,18]. This opens the possibility to close the carbon cycle by re-generating the co-feed via a “power-to-X” process using “green” hydrogen. Although formate is toxic for many organisms even in relatively small concentrations, this is not the case for *U. cynodontis*, which was found to be highly tolerant to the presence of formate in the cultivation medium [1]. Further, studies reported *U. cynodontis* as one of the best itaconate producing species in the family of the Ustilaginaceae, showing relatively high *p*H tolerance in comparison to other smut fungi [4]. Due to its tolerance to low pH values, the broad substrate spectrum comprising various carbohydrate polymers and monomers derived from the degradation of renewable non-food biomass and available genomic tools for potential metabolic engineering approaches, *U. cynodontis* shows great potential as a cell factory for industrial production processes [19]. During previous studies, *U. cynodontis* NBRC 7530 even showed improved itaconate production with sodium formate as a co-substrate in addition to glucose [1]. Sodium formate is believed to be an energy donor via formate dehydrogenase activity rather than a conventional carbon-source [20]. This indicates a putative role of formate delivering extra electrons to the fungal metabolism rather than being converted into biomass or product(s) of interest [21].

**Figure 1:**
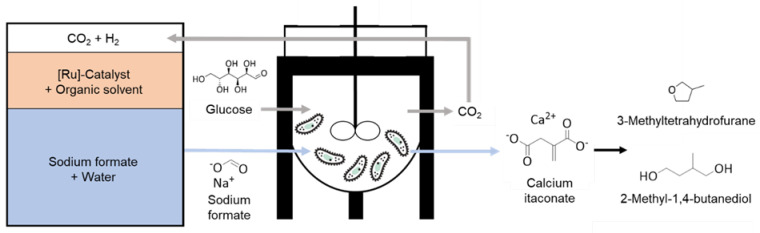
Schematic overview—implementation of CO_2_-derived formate from chemo-catalysis as co-substrate for itaconate production in biocatalysis. In a multiphasic approach with separable and recyclable metal catalyst, CO_2_ is hydrogenated to the respective formate salt. The obtained aqueous product solution from chemo-catalysis is directly applied as a co-feed for the biocatalytic production of itaconate from glucose by Ustilaginaceae. Possible application of itaconic acid for the production, e.g., of biofuels such as 3-methyl-tetrahydrofuran (3−MTHF) and 2−methyl−1,4−butanediol, are depicted [27,28].

While previous work on formate co-metabolism of Ustilaginaceae focused on wild type strains [1], in this study we investigated several available metabolically engineered strains established by Tehrani et al. (2019) for the itaconate production in the presence of formate [3]. By applying the design-of-experiments (DoE) methodology, cultivation parameters (glucose, sodium formate, and ammonium chloride concentrations) were adjusted, enabling further bioprocess optimization towards a continuous production process. In order to demonstrate the possibility for regeneration of the co-feed via a “power-to-X” technology, aqueous solutions of formate were produced via catalytic hydrogenation of CO_2_ and directly used for co-feeding as visualized in Figure 1. Using a biphasic catalytic approach [22,23,24], a tailored Ru-catalyst was “immobilized” in an apolar organic phase [25,26], while the virtually metal- and solvent-free aqueous phase containing the formate salt could be used as co-feed in the fermentation directly, without any further treatment. The biphasic approach not only contributes to the biocompatibility of the product solution but also enables catalyst reusability, which is highly desirable for a sustainable process.

## 2. Materials and Methods

### 2.1. Strains and Culture Conditions

All strains used in this work are listed in Table 1.

Growth and production experiments were performed using a modified Tabuchi medium according to Geiser et al. (2016), containing 0.2 g L^−1^ MgSO_4_·7H_2_O, 0.01 g L^−1^ FeSO_4_·7H_2_O, 0.5 g L^−1^ KH_2_PO_4_, 1 mL L^−1^ vitamin solution, 1 mL L^−1^ trace element solution, and as buffer 19.5 g L^−1^ 2-(N-morpholino) ethanesulfonic acid (MES) or 66 g L^−1^ CaCO_3_ [29]. Different carbon sources such as glucose and sodium formate were used, and the C-source concentrations varied in different experiments. NH_4_Cl was added in indicated concentrations. The vitamin solution contained (per liter) 0.05 g D-biotin, 1 g D-calcium pantothenate, 1 g nicotinic acid, 25 g myo-inositol, 1 g thiamine hydrochloride, 1 g pyridoxol hydrochloride, and 0.2 g para-aminobenzoic acid. The trace element solution contained (per liter) 1.5 g EDTA, 0.45 g ZnSO_4_·7H2O, 0.10 g MnCl_2_·4H_2_O, 0.03 g CoCl_2_·6H_2_O, 0.03 g CuSO_4_·5H_2_O, 0.04 g Na_2_MoO4·2H_2_O, 0.45 g CaCl_2_·2H_2_O, 0.3 g FeSO_4_·7H_2_O, 0.10 g H_3_BO_3_, and 0.01 g KI. Cultivation experiments were performed at 30 °C.

Cultivations in connection with itaconic acid production during the Design of Experiments (DoE) approach were performed in System Duetz^®^ (24 deep-well microtiter plates, EnzyScreen, Heemstede, the Netherlands) with a filling volume of 1.5 mL (300 rpm, 80% humidity, d = 50 mm, Infors HT Multitron Pro shaker, Bottmingen, Switzerland) [30]. Cultures were inoculated in parallel into multiple microtiter plates to a final OD_600_ of 0.5 with cells from an overnight culture in MTM. Tested media combinations are listed in Table 2 For each sample point, a complete plate was taken as a sacrificial sample to ensure continuous oxygenation. Therefore, samples for analytical methods (see Section 2.2) were taken at 5 timepoints distributed throughout the experiment. Experiments were terminated, at the latest, after 678–785 h when a stable itaconate production was observed.

Controlled-fed-batch cultivations were performed in a BioFlo^®^ 120 bioreactor with a total volume of 1.3 L and a working volume of 0.5 L in combination with DASware Control Software 5.3.1 (Eppendorf, Hamburg, Germany). Cultivations were performed in batch medium containing 0.2 g L^−1^ MgSO_4_·7H_2_O, 0.01 g L^−1^ FeSO_4_·7H_2_O, 0.5 g L^−1^ KH_2_PO_4_, 1 g L^−1^ yeast extract, 1 mL L^−1^ vitamin solution, 1 mL L^−1^ trace element solution, and 19.5 g L^−1^ MES as buffer. Various glucose, co-substrate (acetate, formate) and NH_4_Cl concentrations were used. During cultivation, pH was monitored via online pH probes (phferm, Hamilton Company, Bonaduz, Switzerland) and maintained at pH 6.5 by automatic addition of 10 M NaOH and 1 M HCl. Dissolved oxygen tension (DOT) was kept constant at approximately 80% saturation by automatic adjustment of the stirring rate (800–1200 rpm). The bioreactor was aerated with an aeration rate of 1 L min^−1^ (2 vvm), while evaporation was limited by sparging the air through a water bottle. The temperature was set at 30 °C. The bioreactor was inoculated to a final OD_600_ of 0.5 with cells from an overnight culture in 50 mL MTM containing 50 g L^−1^ glucose and 5 g L^−1^ of respective co-substrate.

### 2.2. Analytical Methods

Cell growth was determined by measuring the optical density at 600 nm (OD_600_) with an Ultrospec 10 Cell Density Meter (Amersham Biosciences, Buckinghamshire, UK).

Carbon sources and metabolites such as glucose, acetate, formate, itaconate, malate, succinate and erythritol in the supernatant were analyzed via high-performance liquid chromatography (HPLC). All samples were filtered with Rotilabo^®^ syringe filters (CA, 0.20 µm) and afterward diluted in a range of 1:5–1:50 with 5 mM H_2_SO_4_. Supernatants were analyzed in a DIONEX UltiMate 3000 HPLC System (Thermo Scientific, Waltham, MA, USA) with a Metab-AAC column (300 × 7.8 mm column, ISERA). Elution was performed with 5 mM H_2_SO_4_. at a flow rate of 0.6 mL, min^−1^, and a temperature of 40 °C. For detection, a SHODEX RI-101 detector (Showa Denko Europe GmbH, Munich, Germany) and a DIONEX UltiMate 3000 Variable Wavelength Detector set to 210 nm were used. Analytes were identified via retention time and UV/RI quotient compared to corresponding standards.

All values are the arithmetic mean of three biological replicates. Error bars indicate the standard deviation from the mean. Statistical analysis of significant difference (*p*-value) was performed using unequal variances *t*-test with unilateral distribution (*p* values: <0.01).

^1^H-NMR measurements were conducted at room temperature on a Bruker AS 400 (Bruker Corporation, Billerica, MA, USA) spectrometer. The chemical shift was referenced to the solvent residual signal. Quantitative analysis was conducted using maleic acid as internal standard for aqueous solutions.

### 2.3. Design of Experiments (DoE)

**Figure 2:**
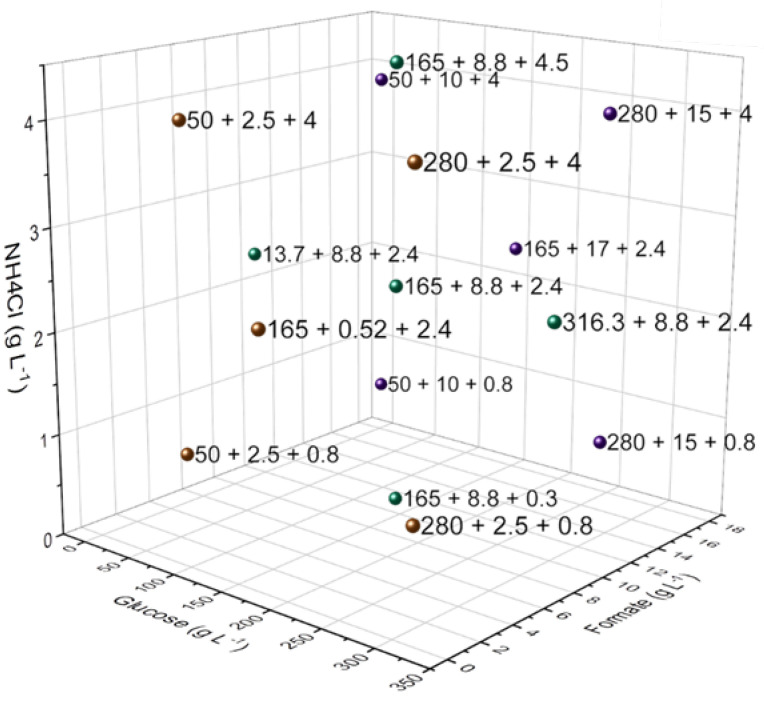
Visualization of the DoE design points. Conditions with the same formate concentration are colored the same way (low concentration colored in orange, medium concentration colored in green and high concentration colored in violet). Conditions of 165 + 8.8 + 2.4 mark the center point. Six points outside the cube are tested as well.

The software Design Expert 11 (Stat-Ease, Minneapolis, MN, USA) was used to set up and evaluate Design of Experiments (DoE) approaches. A response surface 3-factor central composite design (CCD) was chosen to simultaneously evaluate the influence of varying concentrations of glucose, formate and ammonium chloride on itaconic acid production. Fifteen different conditions were tested, whereas the approaches with the lowest and highest glucose concentrations were carried out as quadruplets. All medium level (165 g L^−1^) glucose conditions were implemented as quadruplets too, except the central condition (165 g L^−1^ glucose, 8.75 g L^−1^ formate, 2,4 g L^−1^ ammonium chloride, see Figure 1) as an octuplet. The approaches with 50 and 280 g L-1 glucose were carried out as quintuplets (Table 2). The resulting 72 replicas were split up onto three 24-deep well plates (System Duetz) (Figure 2). The first plate (A) was made up of conditions expected to reach maximum titers within approximately two weeks and the other two plates (B and C) were made up of conditions expected to reach maximum titers within five weeks. The range of factor settings were based primarily on the investigation of single factors during previous studies [1].

**Table 2:** Tested DoE conditions during this study. Different combinations of glucose, formate and ammonium chloride in MTM using CaCO_3_ buffer [29].

No.	Glucose [g L^−1^]	Formate [g L^−1^]	NH_4_Cl [g L^−1^]	CaCO_3_ [g L^−1^]
1	13.7	8.8	2.4	33
2	50	2.5	0.8	
3	50	2.5	4	
4	50	15	0.8	
5	50	15	4	
6	165	0.5	2.4	66
7	165	8.8	0.3	
8	165	8.8	2.4	
9	165	8.8	4.5	
10	165	17	2.4	
11	280	2.5	0.8	
12	280	2.5	4	
13	280	15	0.8	
14	280	15	4	
15	316.3	8.8	2.4	

A quadratic model for predicting the optimal point was chosen and further optimized for optimal values also using Design Expert software. The statistical significance of the model equation was determined by F-value, and the proportion of variance explained by the model obtained was given by the multiple coefficient of determination, R^2^ (ANOVA analysis, see Supplementary Materials).

### 2.4. CO_2_ Hydrogenation in Presence of NaOH and Cis-[RuCl_2_(C_12_-dppm)_2_] in Apolar Solvent/H_2_O

The reagents and solvents were obtained from commercial suppliers. The Schlenk Technique was applied for air-sensitive compounds with an inert argon atmosphere (argon 4.8). The solvents were degassed via a glass frit by bubbling with Argon for 30 min before use. Reaction gases hydrogen 5.0 and carbon dioxide 4.6 were used without further purification.

**Figure 3:**
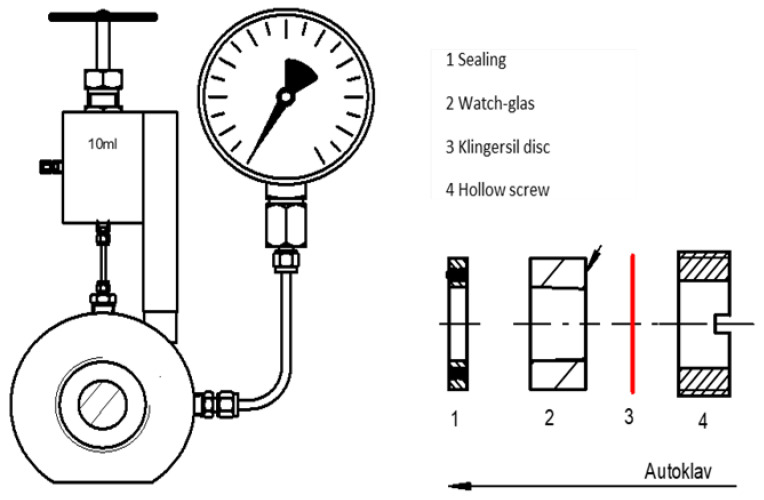
Schematics of the used 10 mL window autoclave.

High pressure reactions were carried out in stainless steel window autoclaves built and maintained by the mechanical workshop of the Institut für Technische und Makromolekulare Chemie of RWTH Aachen University (Figure 3). The autoclaves were equipped with a magnetic stir bar and a digital pressure gauge.

To exclude oxygen from the system, vacuum was applied (<1 × 10^−2^ mbar) in preparation for the experiments, followed by flushing with argon. This procedure was repeated at least three times. An aqueous solution of NaOH (1 M, 3 mL) was introduced in argon counterflow. The catalyst *cis*-[RuCl_2_(C_12_-dppm)_2_] [26] (2.29 mg, 1 µmol) was dissolved in octylacetate, anisole, or tetradecane (2 mL), and added into the autoclave. The autoclave was sealed and pressurized with CO_2_ (30 bar) under stirring to saturate the solution. Then the pressure was released and H_2_ (60 bar) was added. The autoclave was then heated to 70 °C and the pressure monitored with digital pressure gauges connected to a PC. The pressure increased upon heating; when a constant value was reached, the reaction was started by switching on the stirring. Completion of the reaction was indicated by constant pressure after the pressure drop (within 0.5 h with anisole, 2 h with octyl acetate, and 25 h with tetradecane). The autoclave was then cooled to r.t. and the pressure was released carefully. For catalyst recycling, the aqueous phase was removed carefully in argon counterflow and analyzed by ^1^H-NMR as described to determine the formate concentration. To the catalyst phase still residing in the reactor, fresh NaOH-solution was added in argon counterflow. The procedure was then repeated two times as described. The removed aqueous product phases were used in fermentation experiments without any further treatment. The obtained concentration of sodium formate was 0.78–0.81 mol L^−1^, corresponding to 53–55 g L^−1^.

## 3. Results and Discussion

### 3.1. Formate Co-Metabolization with Metabolically Engineered U. cynodontis Strains

**Figure 4:**
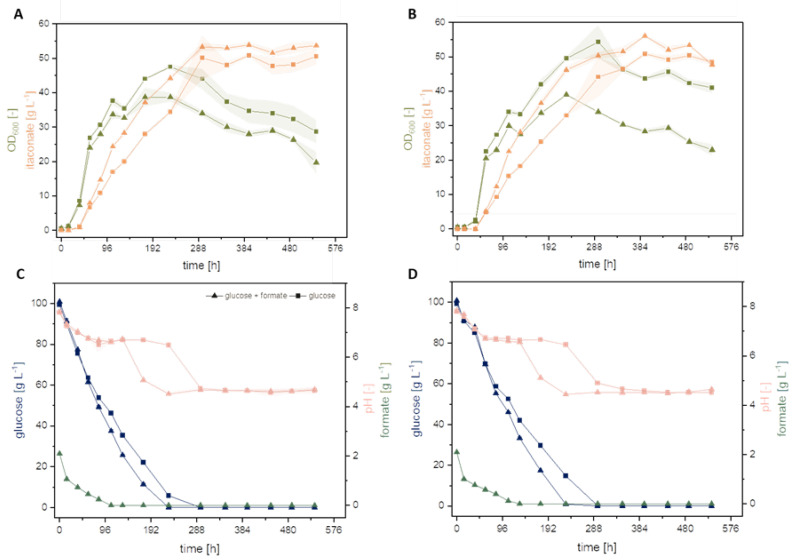
Formate co-metabolization of metabolically engineered U. cynodontis strains. (**A**,**C**) display cultivation of *U. cynodontis* Δ*fuz7* Δ*cyp3* ↑P_ria1_; (**B**,**D**) of *U. cynodontis* Δ*fuz7* Δ*cyp3* P_etef_*mttA* ↑P_ria1_. Formate co-metabolization conditions are shown via ▲ and the respective glucose reference as ■. Color code shows OD_600_ (green), itaconate production (orange), glucose consumption (blue), sodium formate consumption (dark green), pH (light pink). Cultivations were carried out in shake flasks (see Section 2.1). MTM medium (33 g L^−1^ CaCO_3_, 0.8 g L^−1^ NH_4_Cl, 100 g L^−1^ glucose) with 0 g L^−1^ or 2 g L^−1^ sodium formate was used. Error bars depict the standard deviation of the mean (*n* = 3).

Previous experiments showed that glucose, sodium formate, and ammonium chloride (NH_4_Cl) are significant factors impacting the itaconate production of *U. cynodontis* [1,31]. Moreover, recent studies confirmed a beneficial effect of formate co-metabolization on itaconate production by testing 72 different Ustilaginaceae wildtype strains [1]. Among these, *U. cynodontis* #2705 was identified as the most promising strain for metabolizing formate, achieving a 30% increase in itaconate titer in comparison to sole glucose as a carbon source [1]. In contrast to a previous work [1], the study focused on by Tehrani et al. (2019) metabolically engineered *U. cynodontis* strains (*U. cynodontis* Δ*fuz7* Δ*cyp3* ↑P_ria1_ and *U. cynodontis* Δ*fuz7* Δ*cyp3* P*_etef_mttA* ↑P_ria1_) for formate co-metabolization (Figure 4) [3].

The addition of sodium formate had a significant impact on the obtained itaconate titer and yields for both metabolically engineered strains compared to their respective glucose reference (Table 3), as already observed with *U. cynodontis* wild type strains. Both strains displayed a ca. 10% increase in itaconate titer with co-consumption of formate. *U. cynodontis* Δ*fuz7* Δ*cyp3* ↑P_ria1_ reached a maximum itaconate titer of 50.9 ± 0.3 g L^−1^ during cultivation on glucose and a titer of 53.9 ± 0.7 g L^−1^ with the addition of sodium formate. A higher itaconate titer of 56.1 ± 0.2 g L^−1^ with sodium formate co-metabolization (2 g L^−1^) was reached using *U. cynodontis* Δ*fuz7* Δ*cyp3* P*_etef_mttA* ↑P_ria1_, whereas the glucose control led to an itaconate titer of 50.9 ± 0.4 g L^−1^. Together with the improved itaconate titers, the yield increases to 0.55 g_itaconate_ g_substrate_^−1^, compared to 0.51 g_itaconate_ g_glucose_^−1^ without formate.

The results indicate a beneficial effect of sodium formate on metabolically engineered strains similar to the wild type strains tested in previous studies [1]. A potential formate assimilation pathway for *Ustilago* was previously presented by Ullmann et al. (2021) [1]. The improved itaconate production observed during this and previous work could potentially result from extra electrons, which were delivered by formate co-consumption to the fungal metabolism. A further improvement in production may be expected upon parameter optimization, e.g., the feeding ratio of formate to glucose, which is focus of the following study.

### 3.2. Optimization of Formate Co-Metabolization via Design of Experiment (DoE) Approach

In contrast to a one-factor-at-a-time (OFAT) optimization, a DoE approach provides a deeper understanding of how different combinations of the following factors, i.e., glucose, formate, and ammonium chloride, impact itaconate production. Itaconate production will be evaluated by the responses titer and yield. The importance of ammonium chloride for itaconate production was previously shown by Maassen et al. (2014), as *Ustilago* produces itaconate only under nitrogen limitation [31]. Based on previous studies by Tehrani et al. (2019), and the performed shake flasks cultivation within this study, *U. cynodontis* Δ*fuz7* Δ*cyp3* ↑P*_etef_mttA* ↑P*_ria1_* was chosen for the following DoE experiments [8]. Low, medium, and high levels for each parameter (glucose, sodium formate, and ammonium chloride) included in the central composite (CCD) response surface design (RSD) are reported in Table 2 and Table 4. Thereby, 72 experiments were performed to establish CCD models.

**Figure 5:**
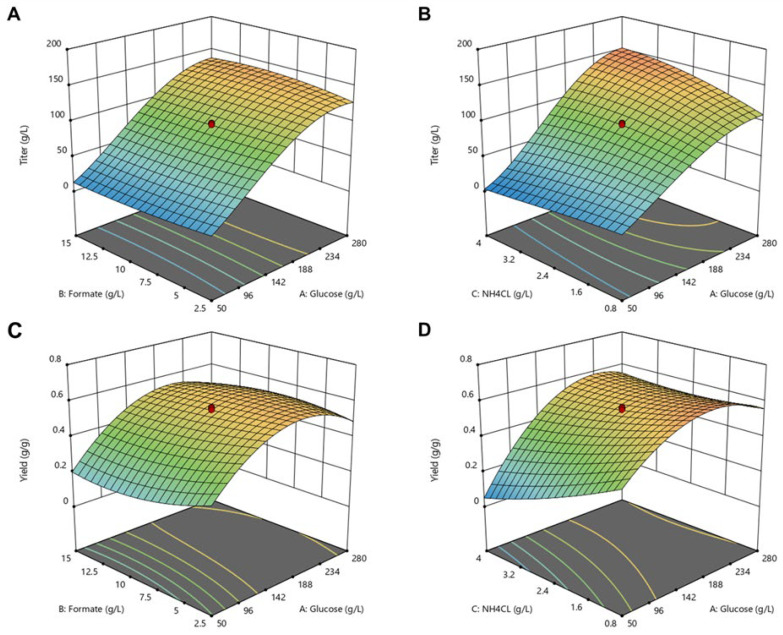
D-surface diagrams of the effect of glucose, formate and ammonium chloride on itaconate production. (**A**,**B**): itaconate titer, (**C**,**D**): itaconate yield; (**B**,**D**) at a sodium formate concentration of 8.8 g L^−1^, (**A**,**C**) at an ammonium chloride concentration of 2.4 g L^−1^. Red dots represent the design points of the model (filled above predicted value, transparent below predicted value). Models were established for *U. cynodontis* Δ*fuz7* Δ*cyp3* P_etef_*mttA* ↑P_ria1_. Color of the diagrams indicate predicted itaconate titer (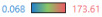 g L^−1^ itaconate) and yield (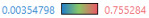 g g^−1^).

The obtained results (Table S2 in Supplementary Materials) furnished the input for the DoE models, one for the titer and one for the yield. The ANOVA analysis confirmed the calculated models to be significant, as implied by the high F- and R^2^-values (Figures S1 and S2 in Supplementary Materials). Thus, the established models show an R^2^-value of 0.9918 for the titer and 0.9845 for yield. Figure 5 displays the influence of formate, glucose and ammonium chloride on the itaconate titer and yield visualized via 3D-surface diagrams.

An increase in the C-source concentration is predicted to result in an increase in the itaconate titer, as indicated by the red color in the heat-map and the increased area of the 3D-surface diagram (Figure 5A,B). An upper limit of glucose concentration, after which the titer drops again, was not determined within the given design space up to 280 g L^−1^ glucose and 15 g L^−1^ sodium formate.

Further, interactions between the model factors are visualized in Figure S6 for itaconate titer. The non-linear correlation of increasing glucose concentrations and itaconate titer can be explained by the fact that all cultures need nitrogen limitation to induce itaconate production after an initial growth phase [32]. A certain amount of glucose is used to build up biomass, until further biomass production is impossible due to nitrogen limitation. The cultures with low glucose concentrations may have lost most of their accessible carbon into biomass, resulting in only a marginal amount of remaining carbon available for itaconate production [33].

Similar trends have been observed for the yield, where low glucose concentrations led to low itaconate yields. However, the highest possible glucose concentration of 280 g L^−1^ did not result in the highest possible yield. In contrast, glucose concentrations in the range of 165 g L^−1^ (in combination with 2.5 g L^−1^ sodium formate and 0.8 g L^−1^ NH_4_Cl) led to the highest predicted yields of 0.67 g g^−1^ (Figures S4 and S5). This optimum glucose concentration for the yield can be attributed to the right balance between higher biomass formation and acceptable osmotic stress. At least for batch cultivations, this sets a limit in efficiently achievable itaconate titers. Fermentations with a continuous feed of glucose could be a viable alternative for reaching high titers with yields above 0.6 g_itaconate_ g_substrate_^−1^ avoiding osmotic stress at the beginning of the process.

Figure S4A–F show the interaction of glucose and sodium formate concentration on itaconate production. It can be observed that the response for itaconate titer did not differ much within the tested range of 2.5 to 15 g L^−1^ sodium formate, and the minimum sodium formate concentration (2.5 g L^−1^) used already provides an increased titer (26.8 ± 0.6 g L^−1^). Nevertheless, the darkest areas of itaconate titers are shown in a range between 5 and 15 g L^−1^ sodium formate (Figure S5B,C). In contrast, the trend of itaconate yield as shown in Figure S4 and Figure 5D–F indicate that low sodium formate concentrations (>5 g L^−1^) are beneficial compared to higher concentrations towards itaconate yield, as darker areas can be found in this range. Results indicate an inhibitory effect of higher formate concentrations on itaconate production. Those toxic effects of formate and product inhibition by higher itaconate concentrations could be addressed in suitable feeding strategies by fed-batch and continuous process approaches. Hosseinpour Tehrani et al. (2019) demonstrated the advantages of a repeated batch approach with cell recycling of *U. cynodontis* [34]. This study showed that the cell recycling positively affected the product yield, which was stable across two repeated batches [34]. During subsequent experiments, an external membrane module could be implemented, enabling cell retention system as repeated batch-cultivations, with *U. cynodontis* already demonstrating high process development potential.

As itaconate production becomes induced upon ammonium limitation, it was expected that the concentration of ammonium chloride, as the sole ammonium supplier of these cultivations, will impact itaconate production during DoE experiments [31]. Thereby, two different effects were observed. Predicted itaconate titer benefits from a lower ammonium chloride concentration (0.8 g L^−1^ NH_4_Cl) when glucose concentration was below 142 g L^−1^ (Figure S6C,D). When higher glucose concentrations are applied, a concentration of 4 g L^−1^ NH_4_Cl improves the predicted titers compared to a low ammonium chloride concentration of 0.8 g L^−1^. As far as the itaconate yield is concerned, lower ammonium chloride concentrations were beneficial for glucose concentrations higher than 234 g L^−1^ (Figure S7).

To sum up, empirical models were generated, which can predict itaconate acid titers and yields within the tested design space at given starting concentrations of glucose, sodium formate, and ammonium chloride. During these experiments, different concentrations of these three substrates were used to gain underlaying data so that the models can accurately predict titer and yields for *U. cynodontis*. The calculated models can be used to optimize the given parameters, maximizing itaconate production.

Those predicted maximum production conditions were applied during cultivation experiments to verify the established models. Two numerical optimizations were performed, resulting in three predictions. Firstly, the best conditions for maximizing itaconate titer, while keeping all factors in range, were predicted for following starting concentrations: glucose 238.8 g L^−1^, sodium formate 9.35 g L^−1^, and ammonium chloride 3.5 g L^−1^. Secondly, the best prediction for maximizing itaconate yield resulted in much lower starting concentrations (glucose 165 g L^−1^, sodium formate 2.5 g L^−1^, and ammonium chloride 0.8 g L^−1^). By chance, these latter conditions were already applied in one of the preliminary experiments for establishing the *DoE* model, and, therefore, cannot be used for model validation. Thirdly, the best prediction for maximizing both responses, while minimizing glucose, foretold following initial concentrations: glucose 95.2 g L^−1^, sodium formate 2.5 g L^−1^, ammonium chloride 0.8 g L^−1^.

**Figure 6:**
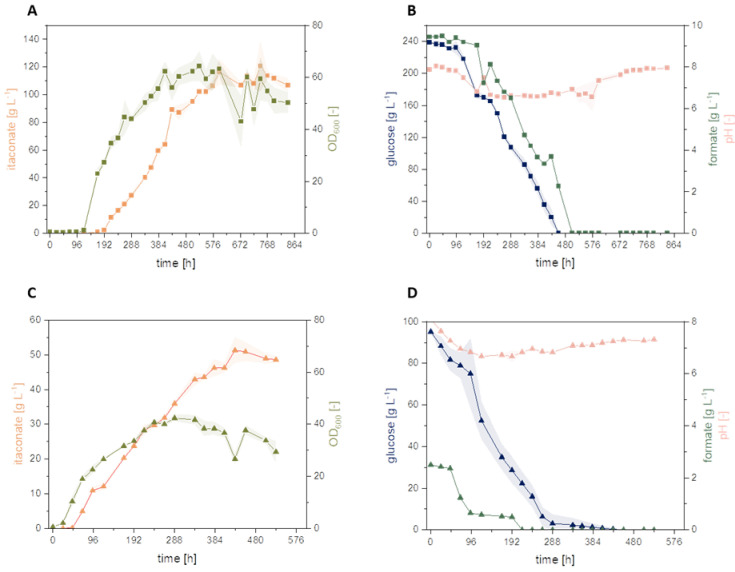
Model validation experiment. Two validation conditions were tested in shake flask cultivation using *U. cynodontis* Δ*fuz7* Δ*cyp3* P_etef_*mttA* ↑P_ria1_. (**A**,**B**) display cultivation using 238.8 g L^−1^ glucose, 9.35 g L^−1^ sodium formate, and 3.5 g L ^−1^ NH_4_Cl (■) while (**C**,**D**) show cultivation using 95.2 g L^−1^ glucose, 2.5 g L^−1^ sodium formate, and 0.8 g L^−1^ NH_4_Cl (▲). Color code shows OD_600_ (light green), itaconate production (orange), glucose consumption (blue), sodium formate consumption (dark green), pH (light pink). Cultivations were carried out in shake flasks following (Section 2.1). MTM medium with 66 g L^−1^ CaCO_3_ was used. Error bars depict the standard deviation of the mean (*n* = 3).

The model predictions and actual values obtained during cultivation experiments are displayed in Table 5. Figure 6 shows the conducted cultivation experiments for model validation. Thereby, both cultivation experiments were successful for model validation, as the itaconate titer and yield were obtained within the 95% confidence interval of the respective prediction. Furthermore, an itaconate titer of 138.2 ± 7.0 g L^−1^ was reached in the experiment at high glucose concentration, representing the highest titer for *U. cynodontis* published so far [34]. During both validation experiments, a yield of 0.55 ± 0.1 g_itaconate_ g_glucose+formate_^−1^ was achieved, which is 76% of the theoretical maximum yield [10]. Excluding sodium formate from the yield calculation, under the assumption that it is not incorporated into the product but just acts as energy donor, a yield of 0.57 ± 0.1 g_itaconate_ g_glucose_^−1^ was achieved.

Thereby, it has to be stated that the proposed role of formate as an energy source for the cell is just assumed here [20,35]. By conducting subsequent ^13^C-labelling experiments using formate as a carbon source, this hypothesis could be confirmed or falsified, and new insights on the potential formate carbon flux in *U. cynodontis* could be gained.

### 3.3. Implementing CO_2_-Derived Formate

Implementation of CO_2_-derived formate obtained via catalytic hydrogenation into the existing bioprocess was investigated. As a formate salt and not “free” formic acid is used in fermentation and a base is needed to shift the endergonic hydrogenation of CO_2_ to formic acid, the combination of these two processes results in a win-win situation. A biphasic organic/aqueous catalytic system was envisaged, with a homogeneous Ru-catalyst residing preferentially in an apolar organic phase. The aqueous solution containing the formate should be used directly in the fermentation, preferably without any purification steps. Moreover, such biphasic approaches allow facile reusability of the metal catalyst.

To enable implementation, the biocompatibility of various organic solvents with *U. cynodontis* cultivation experiments was verified, because the toxicity of potential traces of the solvent transferred due to cross-solubility in the water phase could inhibit the biotechnological conversion. Aqueous solutions saturated with nine different solvents (anisole, ethylacetate, 2-MTHF, *n*-dodecanol, *n*-octanol, *n*-tetradecane, *n*-hexanol, octylacetate, and toluene) were tested for growth using the *U. cynodontis #2705* wild-type strain in Hungate tubes (Figure S8). Thereby, octylacetate, anisole, and tetradecane were selected for subsequent experiments, as these three solvents showed the strongest growth based on cultures turbidity, and were very comparable with the growth observed in a solvent-free reference aqueous solution.

**Figure 7:**
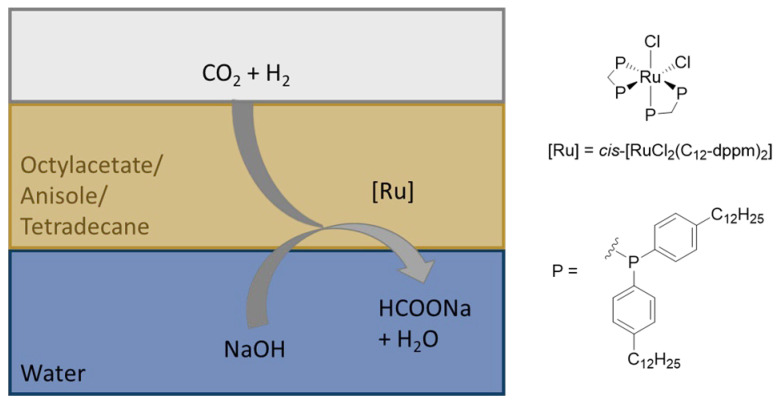
Schematic depiction of the biocompatible, multiphasic system for catalytic conversion of CO_2_ and H_2_ to sodium formate.

The complex *cis*-[RuCl_2_dppm_2_] (dppm = bis(diphenylphosphino)methane) is a well-established catalyst promoting the CO_2_ hydrogenation to formate [23] and was therefore envisaged as lead structure. To ensure good solubility and high retention of the Ru-catalyst in the selected very apolar organic phase, a lipophilic variant of the dppm ligand, the bis(bis(4-dodecylphenyl)phosphanyl)methane (C_12_-dppm), tagged with apolar C_12_ alkyl chains at the phenyl rings was used [22]. With sodium formate chosen as the formate source and the three mentioned solvents as candidates for the catalyst phase, in the next step real product solutions from catalytic hydrogenation of CO_2_ were generated as described in the experimental section and depicted in Figure 7 and Table S3. In a high-pressure reactor, the catalyst phase anisole, octylacetate, or tetradecane (2 mL) containing *cis*-[RuCl_2_(C_12_-dppm)_2_] (2.29 mg, 1 μmol) and an aqueous NaOH solution (1 M, 3 mL) were saturated with CO_2_, simulating gas-scrubbing. The pressure was then released, H_2_ was pressurized (60 bar), and the reactor was heated to 70 °C. The progress of the reaction was monitored through the recorded pressure/time curves (Figure S9). Thus, consumption of gaseous H_2_ leads to a pressure drop and a constant pressure indicates completion of the reaction. Sodium formate solutions with a concentration of 0.78–0.81 M, corresponding to 53–55 g L^−1^ as determined by quantitative ^1^H-NMR, were obtained. Upon separation of the aqueous product phase, the reusability of the catalyst phase was demonstrated for tetradecane, anisole, and octylacetate (Figure S9). Consistent amounts of sodium formate were obtained, as indicated by a similar pressure drop of 10 bar. The reaction rate strongly depends on the used solvent, and reaction completion was observed for anisole after 0.5 h, for octylacetate after 2 h, and for tetradecane after 25 h. However, the reaction rate has a minor importance for this specific application, as the biocompatibility represents the major criterion for the final choice of the solvent. This aspect was examined next.

**Figure 8:**
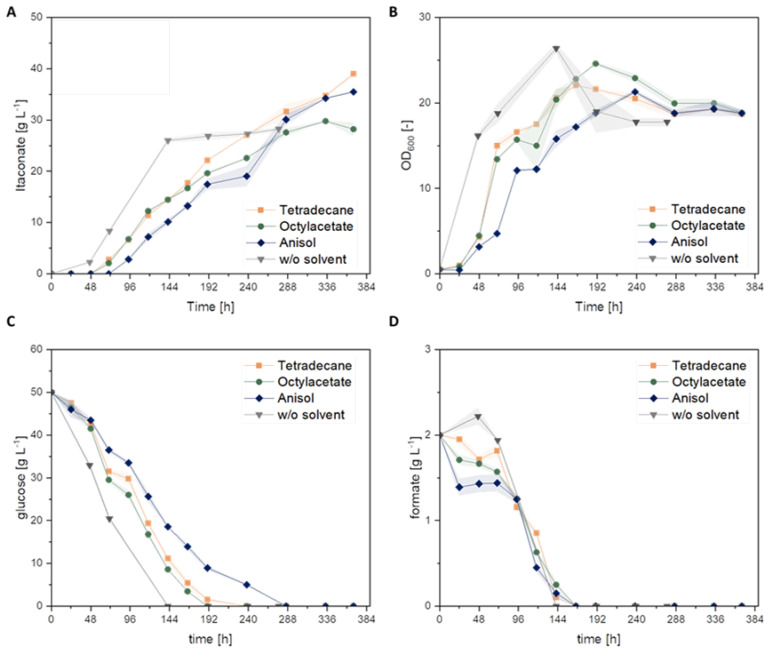
Tolerance of *U. cynodontis* Δ*fuz7* Δ*cyp3* P_etef_*mttA* ↑P_ria1_ towards solvent contamination. (**A**): itaconate production, (**B**): growth via OD_600_, (**C**): glucose consumption and (**D**): formate consumption. Cultivation experiments were performed with 10% filling volume (50 mL) with addition of 1 mL organic solvent using MTM medium with 50 g L^−1^ glucose, 2 g L^−1^ formate, 0.8 g L^−1^ NH_4_Cl. Solvents: tetradecane (■ yellow), octylacetate (● green), anisole (♦ blue) and reference (no solvent, (▼grey). Cultivations were carried out in shake flasks (see Section 2.1). An amount of 33 g L^−1^ CaCO_3_ was used as buffer system. Error bars depict the standard deviation of the mean (*n* = 3).

In a subsequent shake flask cultivation experiment with direct implementation of the formate solutions, the itaconate production was investigated using the metabolically engineered *U. cynodontis* Δ*fuz7* Δ*cyp3 P_etef_mttA ↑P_ria1_* strain displayed in Figure 8 and Table 5. Based on previous cultivations, the product solutions were adjusted to a final formate concentration of 2 g L^−1^ in the cultivation medium, and 50 g L^−1^ glucose was used as a substrate. A prolonged lag phase compared to the previous conditions was observed. This effect could be avoided by adding the materials to the respective pre-culture. The highest itaconate titers of 36.7 ± 0.1 g L^−1^ and 34.3 ± 0.1 g L^−1^ were achieved with the addition of a product formate solution obtained with tetradecane and anisole as catalyst phases, respectively. Thus, the direct use of product solutions originated from the devised biphasic CO_2_ hydrogenation led to an even higher titer as compared with the control experiment with commercial sodium formate solutions (28.4 ± 0.5 g L^−1^). The reason for this marked positive difference is not obvious. No significant impact was observed on cell growth, confirming the biocompatibility of the overall process (Figure 8B).

**Figure 9:**
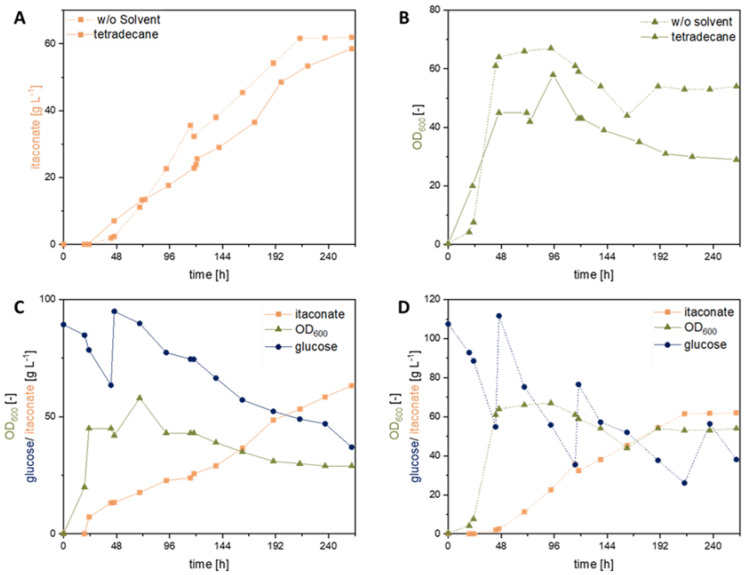
Itaconate production in pulsed fed-batch bioreactor using commercially available sodium formate and formate solutions obtained from biphasic CO_2_ hydrogenation (catalyst phase: tetradecane). *U. cynodontis* Δ*fuz7* Δ*cyp3* P_etef_*mttA* ↑P_ria1_ was used in these experiments. (**A**): itaconate production ■, (**B**): growth via OD_600_ ▲, (**C**): process parameters of cultivation with catalytic product (solid line) and (**D**): cultivation with commercial sodium formate (dashed line). Glucose consumption was visualized via ●. Cultivation was performed with a controlled bioreactor set-up (500 mL filling volume) using MTM medium with 100 g L^−^1 glucose, 4 g L^−^1 sodium formate, 3.5 g L^−^1 NH_4_Cl and MES buffer. pH was controlled at 6.5 via addition of NaOH and HCl (30 °C, 80% DO, *n* = 1). Color scheme is determined as itaconate (orange), OD600 (green), glucose (blue) and formate concentration (yellow).

Based on shake flask cultivation experiments, utilization of sodium formate produced in the catalytic hydrogenation with tetradecane as catalyst phase was further examined during a controlled fed-batch bioreactor experiment (Figure 9). Thereby, culture starting conditions were optimized using the established model. Similar itaconate production titers (Figure 9A) of 63.3 g L^−1^ and 64.2 g L^−1^ were obtained with commercial sodium formate and with CO_2_-derived formate (tetradecane as catalyst phase), Table 6, respectively, in fed-batch experiments. Interestingly, the latter cultivations showed again a significantly higher yield of 0.66 g_itaconate_ g_substrate_^−1^, compared to 0.57 g g^−1^ using commercially available sodium formate.

Based on the promising results obtained during this work using formate product solutions originated from the devised biphasic CO_2_ hydrogenation, further process optimization could be performed by replacing sodium formate by ammonium formate as a product solution obtained via hydrogenation. Thereby, addition of ammonium chloride as a nitrogen source could be minimized. Further, economic evaluation, e.g., via life-cycle assessment (LCA) of the process, could be approached, as the presented work lays the foundation for an improved itaconate production process with a potentially reduced carbon footprint.

## 4. Conclusions

This study demonstrates the use of the pH tolerant and non-filamentous *U. cynodontis* as an alternative itaconate production host in formate co-metabolization experiments. Thereby, an improvement of itaconate production was achieved for metabolically engineered *U. cynodontis* Δ*fuz7* Δ*cyp3* ΔP_ria1_::P_etef_ and *U. cynodontis* Δ*fuz7* Δ*cyp3* ΔP_ria1_::P_etef_ P_etef_*mttA* by the use of sodium formate as co-substrate, increasing production titer (up to 10%) and yield (up to 7%).

Furthermore, *Ustilago* cultivation conditions including ammonium chloride, glucose, and formate co-metabolization were optimized using a DoE approach. Thereby, empirical models were generated, which could predict itaconate titers and yields within the tested design space at given starting concentrations of glucose, formate, and ammonium chloride. Based on the optimized culture conditions, an itaconate titer of 138.2 ± 7.0 g L^−1^ was reached during confirmation experiments corresponding to an increase in maximum titer of 66% with respect to the highest titer for *U. cynodontis* reported so far [32].

The bioprocess could be successfully combined with biphasic catalytic hydrogenation of CO_2_ directly delivering the aqueous sodium formate solutions and closing the carbon cycle for the co-feed. Using a tailored Ru-catalyst in tetradecane as organic phase, the catalyst could be effectively separated from water as the product phase and re-used without loss of activity. The aqueous sodium formate solutions could be directly employed as co-feed for the biotechnological conversion without further purification. Remarkably, even higher titers (+29%) and yields (+28%) were achieved as compared with commercial sodium formate. The yield of up to 0.62 g_itaconate_ g_substrate_^−1^ achieved in this study corresponds to 86% of the theoretical maximum.

## Figures and Tables

**Table 1:** Ustilago cynodontis strains used in this study. Numbers (#) indicate the strain number.

Strain Number	Strain Designation	Resistance	Description	Reference Number
#2705	*Ustilago cynodontis*	wildtype		NBRC 7530
#2706	*Ustilago cynodontis*	wildtype		NBRC 9727
#4852	*Ustilago cynodontis*	carboxin	Δ*fuz7* Δ*cyp3* Δ*P_ria1_::P_etef_*	
#4853	*Ustilago cynodontis*	carboxin	Δ*fuz7* Δ*cyp3* Δ*P_ria1_::P_etef_ + mttA*	

**Table 3:** Itaconate production parameters during shake flask cultivations of metabolically engineered U. cynodontis strains. Cultivations were carried out in shake flasks as described in Section 2.1. MTM medium (33 g L^−1^ CaCO_3_, 0.8 g L^−1^ NH_4_Cl, 100 g L^−1^ glucose) with 0 g L^−1^ or 2 g L^−1^ sodium formate was applied. Error bars depict the standard deviation of the mean (*n* = 3). Statistically significant differences in itaconate production are indicated as * (*p* < 0.05) and ** (*p* < 0.005). Details of statistical analyses are displayed in Table S1.

Condition	Strain	Titer_max_ [g L^−1^]	Y_P/Smax_ [g g^−1^]	q_p_, _max_ [g L^−1^ h^−1^]
100 g L^−1^ glucose 0 g L^−1^ sodium formate 0.8 g L^−1^ NH_4_Cl	*U. cynodontis* Δ*fuz7* Δ*cyp3* ↑P_ria1_	50.9 ± 0.3 *	0.51 ± 0.003	0.12
100 g L^−1^ glucose 2 g L^−1^ sodium formate 0.8 g L^−1^ NH_4_Cl		53.9 ± 0.7 *	0.52 ± 0.007	0.14
100 g L^−1^ glucose 0 g L^−1^ sodium formate 0.8 g L^−1^ NH_4_Cl	*U. cynodontis* Δ*fuz7* Δ*cyp3* P_etef_*mttA* ↑P_ria1_	50.9 ± 0.4 **	0.51 ± 0.004	0.12
100 g L^−1^ glucose 2 g L^−1^ sodium formate 0.8 g L^−1^ NH_4_Cl		56.1 ± 0.2 **	0.55 ± 0.001	0.14

**Table 4:** Factor levels for CCD during this study.

Factor	−1	0	+1
Glucose [g L^−1^]	50	165	280
Sodium formate [g L^−1^]	2.5	8.8	15
Ammonium chloride [g L^−1^]	0.8	2.4	4

**Table 5:** Model validation experiments with *U. cynodontis* Δ*fuz7* Δ*cyp3* P_etef_*mttA*. ± values indicate the standard error of the mean (*n* = 3). Model predictions are highlighted in orange whereas the experimental results are displayed in black. Symbols refer to conditions shown in Figure 6.

Initial Concentrations	Symbol	Titer_max, predicted_	Titer_max_	Y_P/S,max, predicted_	Y_P/S, max_
		[g L^−1^]	[g L^−1^]	[g g^−1^]	[g g^−1^]
238.8 g L^−1^ glucose, 9.35 g L^−1^ sodium formate,	■	*141.9 ± 8.6*	138.2 ± 7.0	*0.57 ± 0.1*	0.55 ± 0.1
3.5 g L^−1^ NH_4_Cl					
95.2 g L^−1^ glucose,	▲	*50.1 ± 5.1*	52 ± 3.3	*0.56 ± 0.1*	0.55 ± 0.1
2.5 g L^−1^ sodium formate,					
0.8 g L^−1^ NH_4_Cl					

**Table 6:** Itaconate production with formate solutions from CO_2_-hydrogenation experiments. Strain *U. cynodontis* Δ*fuz7* Δ*cyp3* P_etef_*mttA* was used. ± Values indicate the standard error of the mean (*n* = 3). Symbols refer to conditions shown in Figure 8 and Figure 9.

Initial Concentrations	Solvent	Symbol	Titer_max_ [g L^−1^]	Y_P/S,max_ [g_ITA_ g_sub_^−1^]
				[g_ITA_ g_glu_^−1^]
50 g L^−1^ glucose, 2 g L^−1^ sodium formate, 0.8 g L^−1^ NH_4_Cl	w/o solvent + commercial HCOONa	▼	28.4 ± 0.5	0.54 ± 0.1 0.56 ± 0.1
	catalyst in tetradecane	■	36.7 ± 0.1	0.69 ± 0.1 0.71 ± 0.1
	catalyst in octylacetate	●	29.8 ± 0.1	0.57 ± 0.1 0.59 ± 0.1
	catalyst in anisole	♦	34.3 ± 0.1	0.66 ± 0.1 0.68 ± 0.1
100 g L^−1^ glucose, 4 g L^−1^ sodium formate, 3.5 g L^−1^ NH_4_Cl	w/o solvent + commercial HCOONa	■	63.3	0.57 0.59
	catalyst in tetradecane	●	64.2	0.66 0.68

## Data Availability

Not applicable.

## Supplementary Materials

The following are available online at https://www.mdpi.com/article/10.3390/jof8121277/s1, Figure S1: ANOVA analysis for the itaconate titer model; Figure S2: ANOVA analysis for the itaconate yield model; Figure S3: Heatmap diagrams of the effect of glucose, formate, and ammonium chloride on itaconate production; Figure S4: 3D-surface diagrams of the effect of glucose, formate, and ammonium chloride on itaconate production; Figure S5: Heatmap diagrams of the effect of glucose, formate, and ammonium chloride on itaconate production; Figure S6: Interaction between glucose, formate, and ammonium chloride on itaconate titer obtained via DoE approach; Figure S7: Interaction between glucose, formate, and ammonium chloride on itaconate yield obtained via DoE approach; Figure S8: Organic solvent tolerance test in U. cynodontis #2705; Figure S9: Pressure-drop curve of the CO_2_ hydrogenation in presence of NaOH and cis-[RuCl_2_(C1_2_-dppm)_2_] in apolar solvent/H_2_O with A: tetradecane, B: octylacetate, and C: anisole as catalyst phase; Table S1: Welch *t*-test result for shake flask cultivations of metabolically engineered *U. cynodontis* strains; Table S2: Tested DoE conditions and responses during this study; Table S3: Results from the CO_2_ hydrogenation in presence of NaOH and cis-[RuCl_2_(C1_2_-dppm)_2_] in apolar solvent/H_2_O.
