# Supplementary material for: Improved Itaconate Production with Ustilago cynodontis via Co-Metabolism of CO2-Derived Formate
Source: J Fungi (Basel). 2022 Dec 5;8(12):1277. doi: 10.3390/jof8121277 (PMC9784962; doi:10.3390/jof8121277)
Supplement: Supplementary file 1 [file jof-08-01277-s001.zip › jof-2034801-Supplementary Materials.pdf]

Supplemental data to:

Improved Itaconate Production with *U. cynodontis* via Co-Metabolism of CO_2-derived Formate

Lena Ullmann[1], Nils Guntermann[2], Philipp Kohl[1], Gereon Schröders[1], Andreas Müsgens[1], Giancarlo Franciò[2], Walter Leitner[2,3], Lars M Blank[1]*

*to whom correspondence should be addressed

Address 1: [1] iAMB – Institute of Applied Microbiology

ABBt – Aachen Biology and Biotechnology

RWTH Aachen University

Worringerweg 1

D-52074 Aachen, Germany

Address 2: [2]Institute of Technical and Macromolecular Chemistry

Worringerweg 2

D-52074 Aachen, Germany

Adress 3: [3]Max Planck Institute for Chemical Energy Conversion

Stiftstraße 34-36

D-45470 Mülheim an der Ruhr, Germany

Lena Ullmann (lena.ullmann@rwth-aachen.de)

Nils Guntermann (Guntermann@itmc.rwth-aachen.de)

Philipp Kohl (philipp.kohl@rwth-aachen.de)

Gereon Schröders (gereon.schroeders@rwth-aachen.de)

Andreas Müsgens (andreas.muesgens@rwth-aachen.de)

Giancarlo Francío (francio@itmc.rwth-aachen.de)

Walter Leitner (walter.leitner@cec.mpg.de)

Blank, Lars M. (lars.blank@rwth-aachen.de)

Supplementary figures

1. *DoE optimum glucose-formate feeding ratio*

A

ANOVA

Response 2: Titer
Transform: Square Root
Constant: 0.5

Source	Sum of Squares	df	Mean Square	F-value	p-value	
**Model**	951.01	13	73.15	538.27	< 0.0001	significant
A-Glucose	230.84	1	230.84	1698.50	< 0.0001	
B-Formate	0.0674	1	0.0674	0.4957	0.4842	
C-NH4CL	0.9594	1	0.9594	7.06	0.0102	
AB	4.33	1	4.33	31.88	< 0.0001	
AC	54.62	1	54.62	401.91	< 0.0001	
BC	1.28	1	1.28	9.44	0.0032	
A^2	98.14	1	98.14	722.14	< 0.0001	
B^2	0.0338	1	0.0338	0.2487	0.6199	
C^2	3.67	1	3.67	26.97	< 0.0001	
ABC	1.13	1	1.13	8.34	0.0054	
A^2B	0.0531	1	0.0531	0.3906	0.5344	
A^2C	2.65	1	2.65	19.49	< 0.0001	
AB^2	1.36	1	1.36	9.98	0.0025	
**Residual**	7.88	58	0.1359			
Lack of Fit	0.0563	1	0.0563	0.4103	0.5244	not significant
Pure Error	7.83	57	0.1373			
**Cor Total**	958.89	71				

B

Fit Statistics

**Std. Dev.**	0.3687	**R^2**	0.9918
**Mean**	8.00	**Adjusted R^2**	0.9899
**C.V. %**	4.61	**Predicted R^2**	0.9869
		**Adeq Precision**	69.6685

C

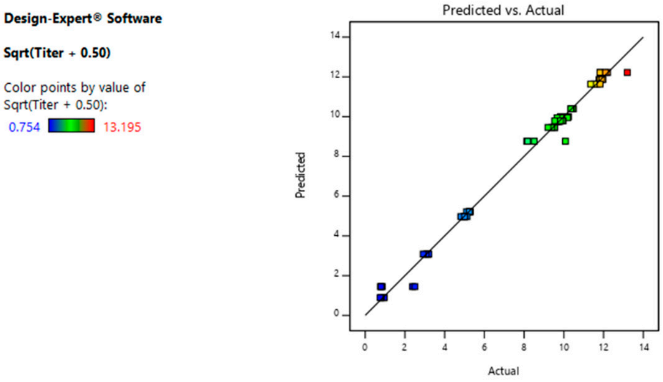

Figure S1. ANOVA analysis for the itaconate titer model. (A) ANOVA analysis table, (B) fit statistics, (C) predicted vs. actual plot.

A

ANOVA

Response 1: Yield

Source	Sum of Squares	df	Mean Square	F-value	p-value	
**Model**	3.33	13	0.2565	282.91	< 0.0001	significant
A-Glucose	0.5079	1	0.5079	560.30	< 0.0001	
B-Formate	0.0013	1	0.0013	1.43	0.2372	
C-NH4CL	0.0075	1	0.0075	8.31	0.0055	
AB	0.0572	1	0.0572	63.14	< 0.0001	
AC	0.5783	1	0.5783	637.90	< 0.0001	
BC	0.0000	1	0.0000	0.0141	0.9058	
A^2	0.6212	1	0.6212	685.22	< 0.0001	
B^2	0.0000	1	0.0000	0.0256	0.8735	
C^2	0.0026	1	0.0026	2.90	0.0940	
A^2B	0.0085	1	0.0085	9.33	0.0034	
A^2C	0.1236	1	0.1236	136.38	< 0.0001	
AB^2	0.0785	1	0.0785	86.57	< 0.0001	
A^2B^2	0.0204	1	0.0204	22.48	< 0.0001	
**Residual**	0.0526	58	0.0009			
Lack of Fit	0.0026	1	0.0026	2.93	0.0924	not significant
Pure Error	0.0500	57	0.0009			
**Cor Total**	3.39	71				

B **Fit Statistics**

**Std. Dev.**	0.0301	R^2	0.9845
**Mean**	0.5279	**Adjusted** R^2	0.9810
**C.V. %**	5.70	**Predicted** R^2	0.9758
		**Adeq Precision**	52.3154

C

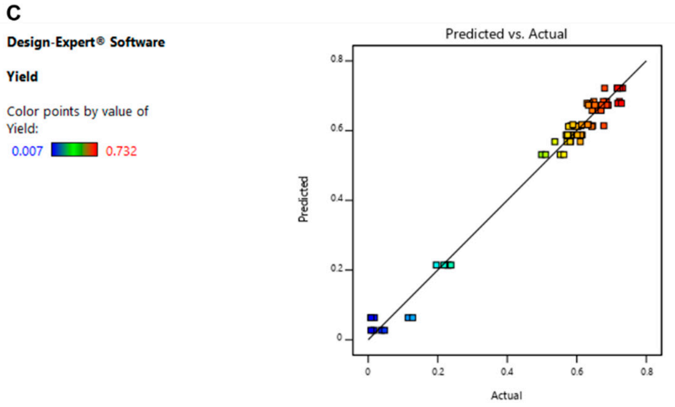

Figure S2. ANOVA analysis for the itaconate yield model. (A) ANOVA analysis table, (B) fit statistics, (C) predicted vs. actual plot.

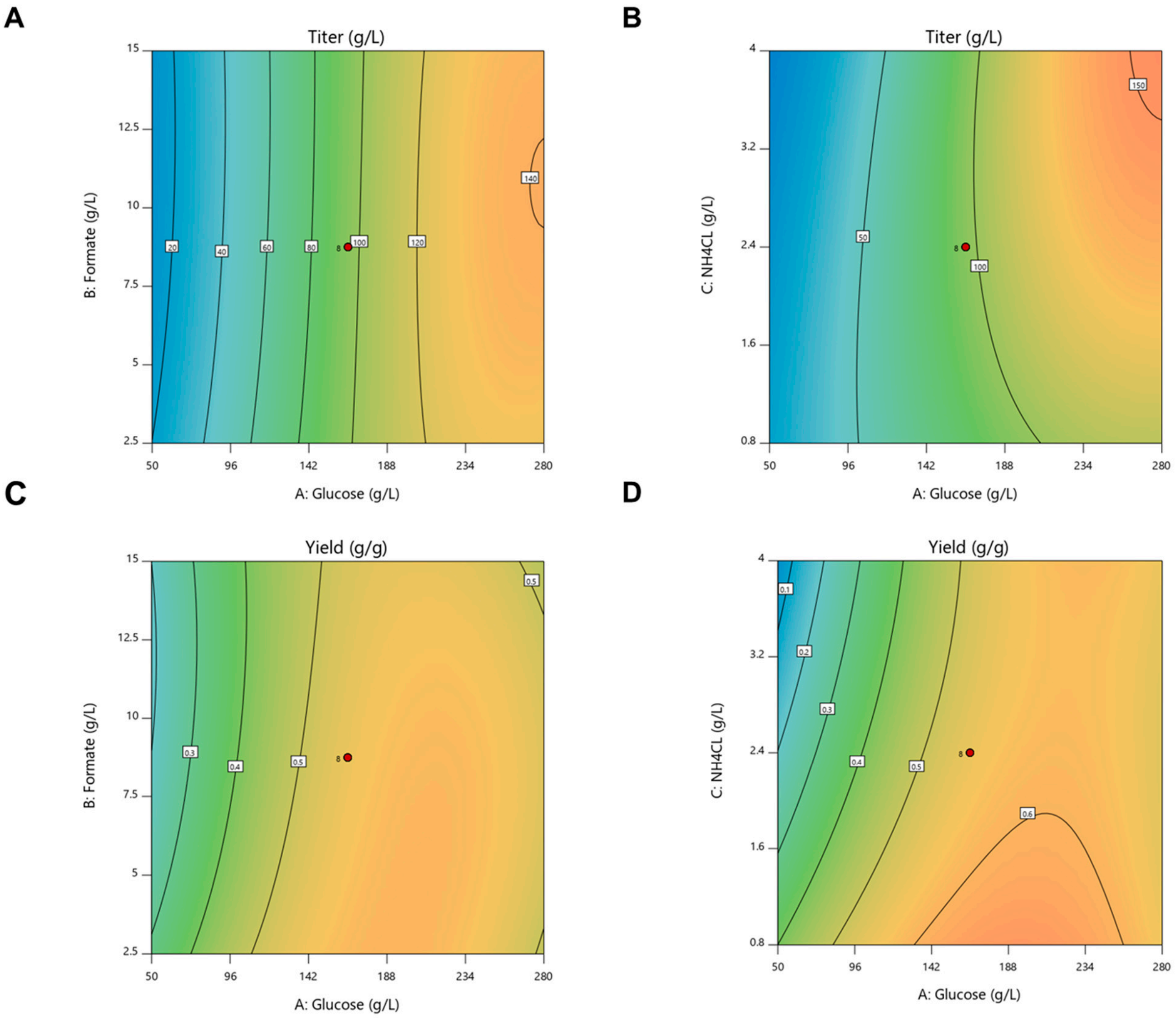

Figure S3 Heatmap diagrams of the effect of glucose, formate and ammonium chloride on itaconate production. A – B: itaconate titer, C - D: itaconate yield with a formate concentrations of 8.8 g L^{-1} (B, D) and an ammonium chloride concentration of 2.4 g L^{-1} (A, C). Red dots represent the design points of the respective model. Models were established for *U. cynodontis Δfuz7 Δcyp3* ↑P_{etef}*mttA* ↑P_{ria1}.

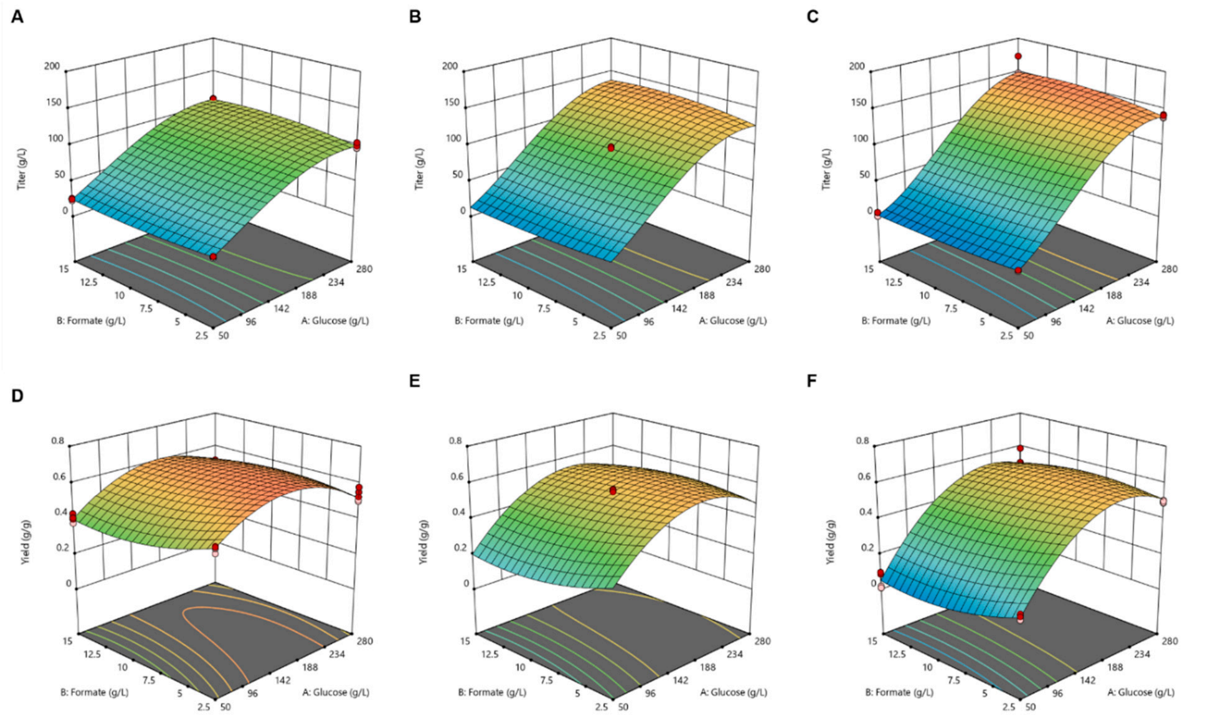

Figure S4 3D-surface diagrams of the effect of glucose, formate and ammonium chloride on itaconate production. A – C: itaconate titer, D-F: itaconate yield with varying ammonium chloride concentrations (A, D: 0.8 g L^{-1}; B, E: 2.4 g L^{-1}; E, F: 4 g L^{-1}). Red dots represent the design points of the model (filled above predicted value, transparent below predicted value). Models were established for *U. cynodontis Δfuz7 Δcyp3* ↑P_{etef}*mttA* ↑P_{ria1}. Color of the diagrams indicate predicted itaconate titer (0.068 – 173.61 g L^{-1} itaconate) and yield (0.00354798 – 0.755284 g g^{-1}).

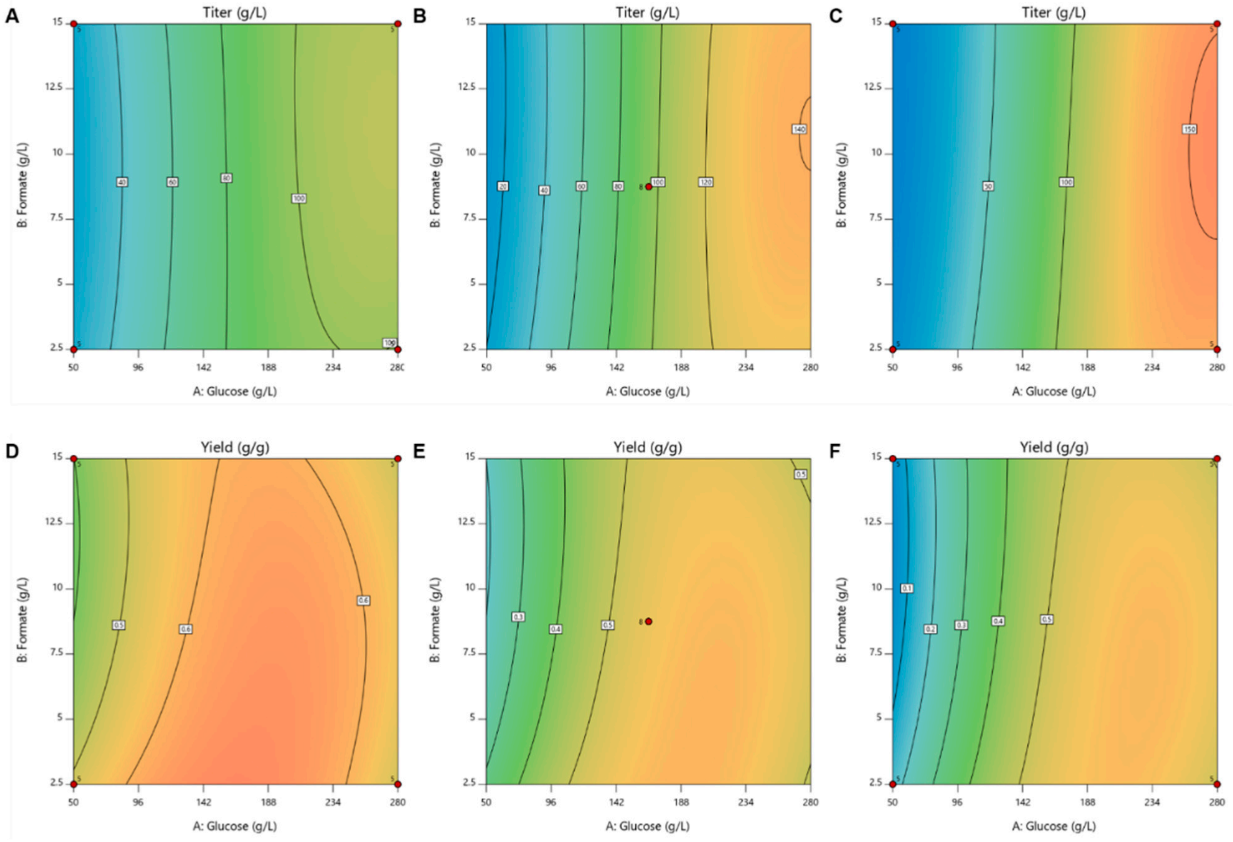

Figure S5 Heatmap diagrams of the effect of glucose, formate and ammonium chloride on itaconate production. A – C: itaconate titer, D-F: itaconate yield with varying NH_4Cl concentrations (A, D: 0.8 g L^{-1}; B, E: 2.4 g L^{-1}; E, F: 4 g L^{-1}). Red dots represent the design points of the respective model. Models were established for *U. cynodontis Δfuz7 Δcyp3* ↑P_{etef}*mttA* ↑P_{ria1}.

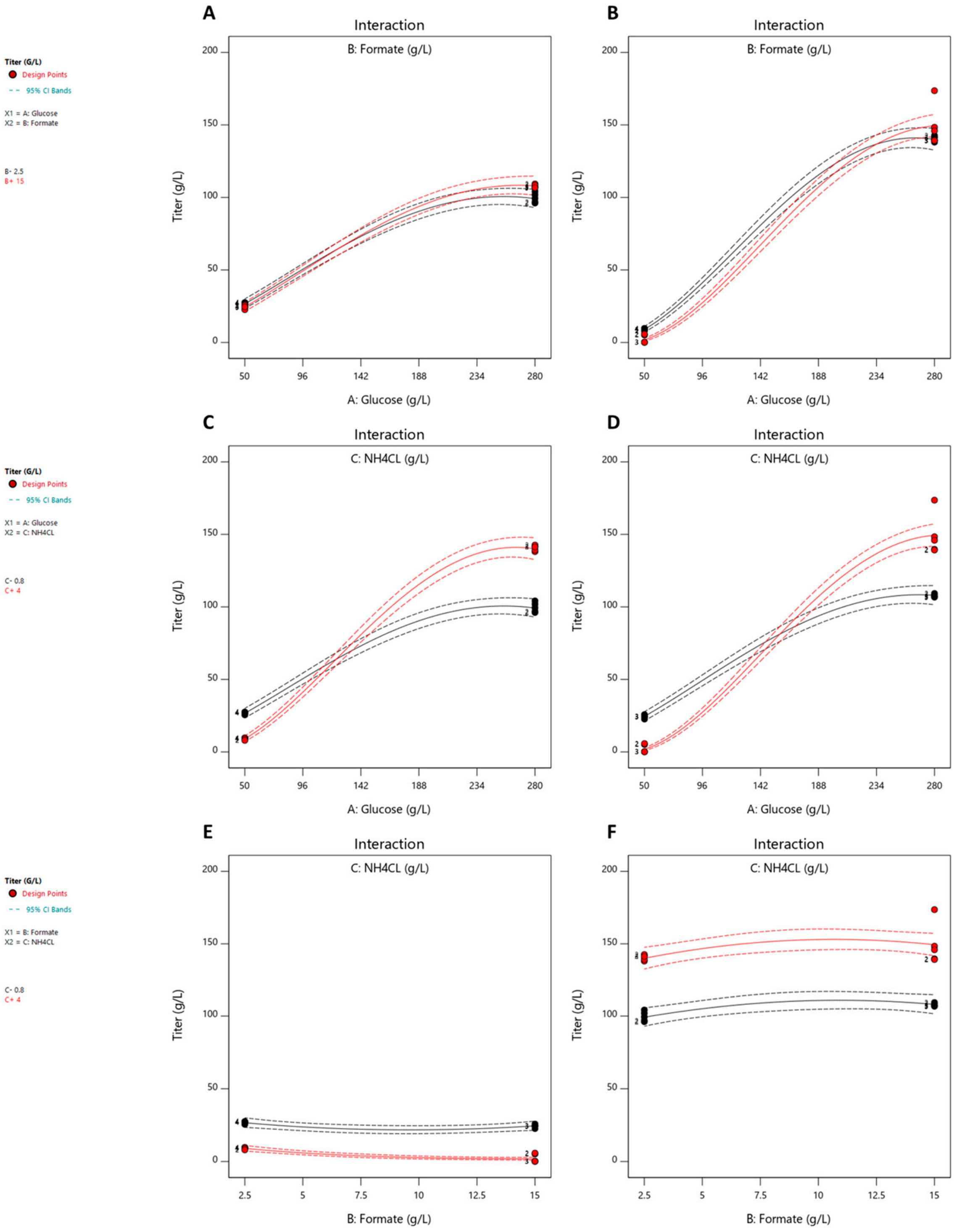

Figure S6 Interaction between glucose, formate and ammonium chloride on itaconate titer obtained via DoE approach. A, B shows interaction of glucose and formate for A: 0.8 g L^{-1} and B: 4 g L^{-1} ammonium

chloride concentrations. C, D shows interaction of glucose and ammonium chloride for C: 2.5 g L^{-1} and D: 15 g L^{-1} formate concentrations. E, F shows interaction of formate and ammonium chloride concentration for E: 50 g L^{-1} and F: 280 g L^{-1} glucose concentrations.

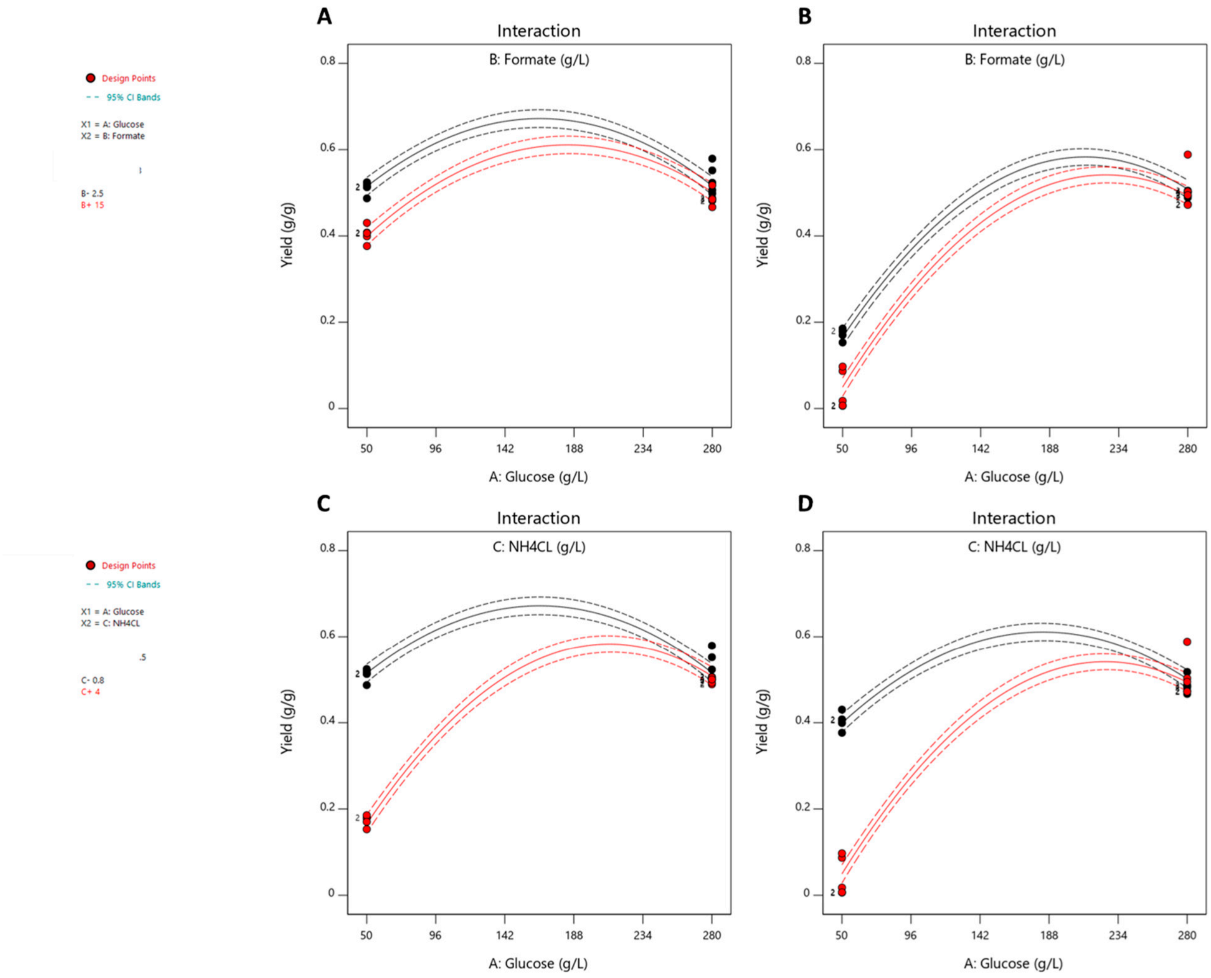

Figure S7 Interaction between glucose, formate and ammonium chloride on itaconate yield obtained via DoE approach. A, B shows interaction of glucose and formate for A: 0.8 g L^{-1} and B: 4 g L^{-1} ammonium chloride concentrations. C, D shows interaction of glucose and ammonium chloride for C: 2.5 g L^{-1} and D: 15 g L^{-1} formate concentrations.

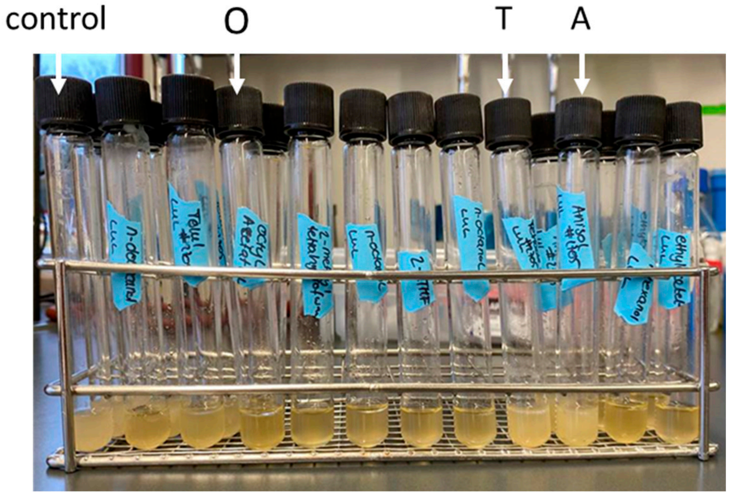

Figure S8 Organic solvent tolerance test in *U. cynodontis* #2705. Experiment was performed using 2 ml YEP-medium, 4 gL-1 glucose in addition of 100 µl solvent (n=2). Control without addition of solvent is displayed on the left. Growth was evaluated based on cultures turbidity. Thereby, ocytlacetate (O), anisol (A) and tetradecane (T) were selected for subsequent experiments.

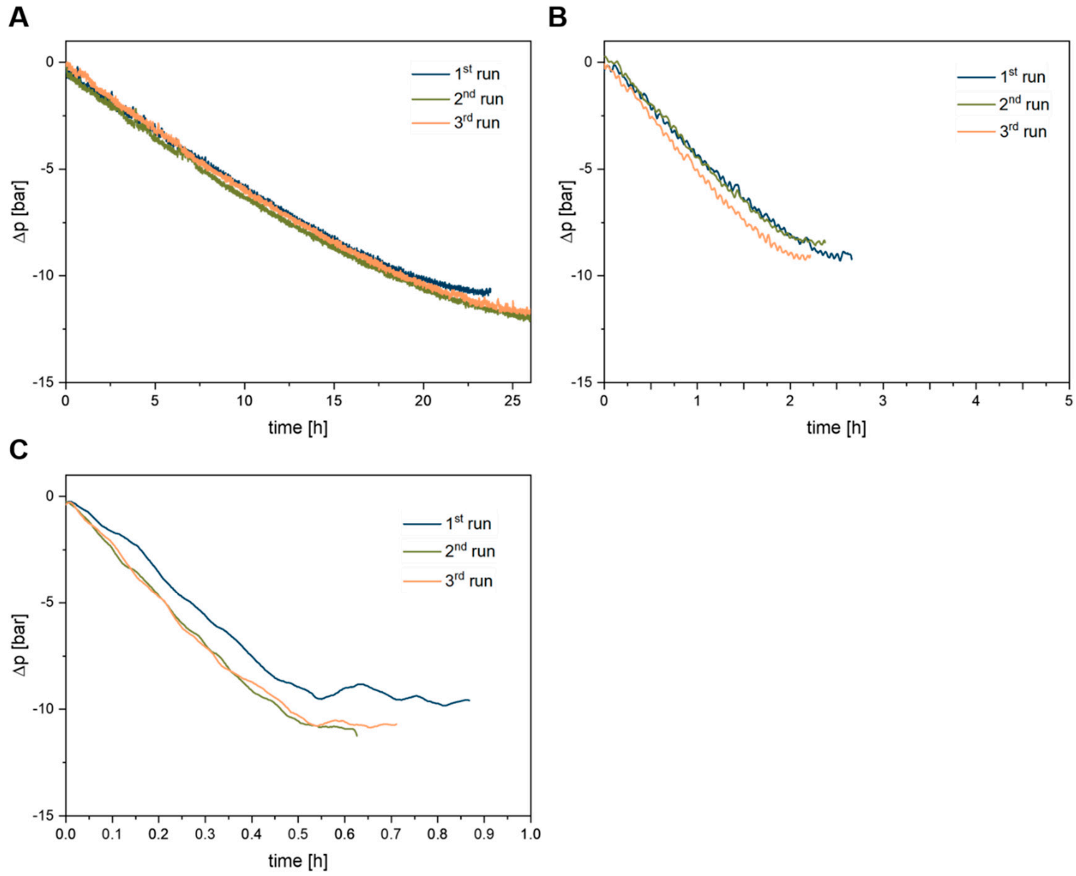

Figure S9 Pressure-drop curve of the CO_2 hydrogenation in presence of NaOH and *cis*-[RuCl$_2$(C$_{12}$-dppm)$_2$] in apolar solvent/ H_2O with A: tetradecane, B: octylacetate and C: anisole as catalyst phase.

Supplementary tables

2. *DoE optimum glucose-formate feeding ratio*

Table S1 Welch t-test result for *shake flask cultivations of* metabolically engineered *U. cynodontis* strains. *#4852: U. cynodontis Δfuz7 Δcyp3* ↑*P*$_{ria1}$ (highlighted in yellow) and *#4853: U. cynodontis Δfuz7 Δcyp3 P*$_{etef}$*mttA* ↑*P*$_{ria1}$ (highlighted in blue*)*. Cultivations were carried out following (**section 4.1**). MTM medium (33 g · L^{-1} CaCO3, 0.8 g · L^{-1} NH_4Cl, 100 g · L^{-1} glucose) with 0 g · L^{-1} or 2 g · L^{-1} formate was used.

t-test: two-sample assuming unequal variances			t-test: two-sample assuming unequal variances		
#4853 maximum titer		shake flasks	#4853 maximum yield		shake flasks
	Glc	*Glc + Form*		*Glc*	*Glc + Form*
Mean	50.866	56.0923333	Mean	0.51234375	0.55606885
Variance	0.277263	0.04606233	Variance	2.8129E-05	4.5268E-06
Observations	3	3	Observations	3	3
Hypothesized Mean Difference	0		Hypothesized Mean Difference	0	
df	3		df	3	
t Stat	-15.9198095		t Stat	-13.2528308	
P(T<=t) one-tail	0.00026946		P(T<=t) one-tail	0.00046418	
t Critical one-tail	2.35336343		t Critical one-tail	2.35336343	
P(T<=t) two-tail	0.00053892		P(T<=t) two-tail	0.00092835	
t Critical two-tail	3.18244631		t Critical two-tail	3.18244631	

t-test: two-sample assuming unequal variances			t-test: two-sample assuming unequal variances		
#4852 maximum titer		shake flasks	#4852 maximum yield		shake flasks
	Glc	*Glc + Form*		*Glc*	*Glc + Form*
Mean	50.859	53.875	Mean	0.51227324	0.53408742
Variance	0.170443	0.674719	Variance	1.7292E-05	6.6309E-05
Observations	3	3	Observations	3	3
Hypothesized Mean Difference	0		Hypothesized Mean Difference	0	
df	3		df	3	
t Stat	-5.68227154		t Stat	-4.13231314	
P(T<=t) one-tail	0.00540075		P(T<=t) one-tail	0.01285643	
t Critical one-tail	2.35336343		t Critical one-tail	2.35336343	
P(T<=t) two-tail	0.0108015		P(T<=t) two-tail	0.02571287	
t Critical two-tail	3.18244631		t Critical two-tail	3.18244631	

Table S2 Tested DoE conditions and responses during this study. Different combinations of glucose, formate and ammonium chloride in MTM using $CaCO_3$ buffer [1]. [a]yield calculation incl. formate, [b]yield calculation excluding formate.

No.	Run	Glucose [g L^{-1}]	Formate [g L^{-1}]	NH_4Cl [g L^{-1}]	Yield [g g^{-1}][a]	Yield [g g^{-1}][b]	Titer [g L^{-1}]
44	10	13.65	8.75	2.4	0.01	0.01	0.07
42	14	13.65	8.75	2.4	0.02	0.03	0.42
43	68	13.65	8.75	2.4	0.01	0.01	0.14
41	72	13.65	8.75	2.4	0.02	0.02	0.34
1	9	50	2.5	0.8	0.49	0.51	25.58
25	25	50	2.5	4	0.16	0.16	8.04
3	32	50	2.5	0.8	0.51	0.53	26.93
22	39	50	2.5	4	0.18	0.18	9.42
4	54	50	2.5	0.8	0.51	0.55	27.25
5	56	50	2.5	0.8	0.01	0.53	26.98
24	60	50	2.5	4	0.17	0.18	8.94
2	64	50	2.5	0.8	0.52	0.55	27.53
21	66	50	2.5	4	0.17	0.18	9.43
23	70	50	2.5	4	0.18	0.19	9.74
14	16*	50	15	0.8	0.37	0.45	22.67
13	18*	50	15	0.8	0.39	0.48	24.17
32	21*	50	15	4	0.02	0.01	0.10
31	31*	50	15	4	0.01	0.01	0.25
12	33*	50	15	0.8	0.40	0.49	24.58
33	34*	50	15	4	0.10	0.10	5.18
34	41*	50	15	4	0.10	0.11	5.76
35	47*	50	15	4	0.01	0.01	0.13
15	51*	50	15	0.8	0.40	0.49	24.54
11	65	50	15	0.8	0.43	0.51	25.76
50	29	165	0.52	2.4	0.62	0.62	103.38
51	37	165	0.52	2.4	0.58	0.58	96.74
49	42	165	0.52	2.4	0.59	0.59	98.05
52	57	165	0.52	2.4	0.56	0.56	92.80
66	1	165	8.75	2.4	0.57	0.59	98.75
62	5	165	8.75	4.5	0.52	0.54	90.29
72	19	165	8.75	2.4	0.52	0.58	96.03
63	24	165	8.75	4.5	0.51	0.53	87.81
57	26*	165	8.75	0.29	0.65	0.65	101.10
71	27	165	8.75	2.4	0.56	0.59	98.01
70	28	165	8.75	2.4	0.52	0.55	90.57
67	30	165	8.75	2.4	0.56	0.59	97.38
64	36	165	8.75	4.5	0.48	0.51	84.20
59	38*	165	8.75	0.29	0.65	0.66	66.35
69	40	165	8.75	2.4	0.56	0.59	97.88
65	43	165	8.75	2.4	0.57	0.60	98.31
68	46	165	8.75	2.4	0.53	0.56	93.22
58	59*	165	8.75	0.29	0.65	0.65	65.66
60	61*	165	8.75	0.29	0.66	0.43	71.95
61	62	165	8.75	4.5	0.52	0.54	90.56
54	2	165	16.98	2.4	0.52	0.57	94.67
53	7	165	16.98	2.4	0.52	0.57	93.86
55	52	165	16.98	2.4	0.51	0.56	92.120
56	63	165	16.98	2.4	0.53	0.58	95.78
9	3*	280	2.5	0.8	0.58	0.58	104.16
28	12	280	2.5	4	0.49	0.49	139.21
29	13	280	2.5	4	0.50	0.50	142.67

10	17*	280	2.5	0.8	0.51	0.51	96.84
8	22*	280	2.5	0.8	0.50	0.50	96.16
27	44	280	2.5	4	0.49	0.49	138.14
7	50*	280	2.5	0.8	0.52	0.53	99.37
30	53	280	2.5	4	0.50	0.50	141.36
26	55	280	2.5	4	0.49	0.50	141.64
6	71*	280	2.5	0.8	0.55	0.55	101.9
37	4	280	15	4	0.47	0.49	139.25
36	8	280	15	4	0.47	0.50	139.67
16	15	280	15	0.8	0.52	0.55	109.29
20	20*	280	15	0.8	0.48	0.49	106.72
39	23	280	15	4	0.50	0.53	148.39
18	35*	280	15	0.8	0.48	0.38	107.08
19	45*	280	15	0.8	0.48	0.51	108.4
38	48	280	15	4	0.59	0.62	173.61
40	67	280	15	4	0.49	0.52	145.95
17	69*	280	15	0.8	0.49	0.49	107.3
48	6*	316.35	8.75	2.4	0.43	0.43	128.31
46	11*	316.35	8.75	2.4	0.42	0.42	132.67
47	49*	316.35	8.75	2.4	0.47	0.48	139.35
45	58*	316.35	8.75	2.4	0.46	0.47	136.12

Table S3 Results from the CO_2 hydrogenation in presence of NaOH and *cis*-[$RuCl_2(C_{12}$-dppm$)_2$] in apolar solvent/ H_2O.

No.	Run	Solvent	Time [h]	c(Formate) [M]	c(Formate) [g L^{-1}]
1	1	Tetradecane	23.7	0.783	53.2
2	2	Tetradecane	26.0	0.788	53.6
3	3	Tetradecane	26.0	0.720	49.0
4	1	Octylacetate	2.7	0.783	53.3
5	2	Octylacetate	2.4	0.793	54.0
6	3	Octylacetate	2.2	0.779	53.0
7	1	Anisole	0.6	0.772	52.5
8	2	Anisole	0.5	0.688	46.8
9	3	Anisole	0.5	0.770	52.4
